# Genetic variations in patient with Parry–Romberg syndrome

**DOI:** 10.1038/s41598-023-27597-1

**Published:** 2023-01-09

**Authors:** Bao-Fu Yu, Li-Ping Dong, Chuan-Chang Dai, Jiao Wei

**Affiliations:** Department of Plastic and Reconstructive Surgery, Shanghai Ninth People’s Hospital Affiliated to Shanghai Jiaotong University School of Medicine, No. 639 Zhi Zao Ju Road, Shanghai, 200011 China

**Keywords:** Skin diseases, Skin manifestations

## Abstract

Parry–Romberg syndrome is a rare craniofacial disorder which is characterized by progressive facial atrophy. The etiology and pathogenesis of the disease are not known. Herein, we report the genetic variants in patient with this disease. A 25-year-old woman was diagnosed with Parry–Romberg syndrome according to her clinical manifestation, which presented with typical progressive unilateral facial soft tissue atrophy. Using peripheral blood samples, Whole exome sequencing (WES) was conducted on this patient and her parents. Variant loci of the genes were validated by Sanger sequencing in her twin sister who had no Parry–Romberg syndrome. Subsequently, we searched the GeneCards^®^: the Human Gene Database for variant genes, annotated them and analyzed their functions. The results of WES showed that 2 genes (*MTOR, DHX37*) were mutated, and the variant loci were *MTOR*: NM_004958.4: exon31: c.4487A>T: p.Q1496L and *DHX37*: NM_032656.4: exon17: c.2180C>T: p.T727M, respectively. However, the variant loci were also detected in her twin sister by Sanger sequencing. The Human Gene Database for variant genes shows that the two genes may be associated with craniomaxillofacial developmental abnormalities. Although *MTOR* and *DHX37* genes were tested and found to have mutations in patient with Parry–Romberg syndrome, these variants may not directly determine the clinical phenotype. When studying clinical etiology, other factors, such as the environment, should also be taken into account.

## Introduction

Parry–Romberg syndrome, also named progressive hemifacial atrophy, was first reported by Parry in 1824 and Romberg in 1846^1^. This is a rare disease, with symptoms manifested as atrophy of the skin, subcutaneous tissue, fat, and even muscles and bones of the hemifacial^2,3^. As it is a rare disease, no incidence of the disease has been reported. Most of the previous literatures reported several cases of hemifacial atrophy, among which one study reported 16 cases of hemifacial atrophy, but their family genetic information was not reported in detail^12^. So it is not known whether the disease runs in families. Although the age of onset varies among patients, most patients have onset within the age of 10 or within the age of 20 years^4^. The male to female incidence rate is 1:1.5. The disease has a self-limiting period of 2–10 years before stabilization^5^. The clinical presentation is very similar to linear scleroderma en coup de sabre^6^. It is also possible for both diseases to coexist, making it very difficult to distinguish between them^7^. Extracutaneous disease manifestations, including neurological, ocular, and oral pathology, are common and may occur at any stage of the disease. At present, the severity of the disease is usually divided into mild, moderate and severe to guide treatment according to the range of involvement of sensory branch of the trigeminal nerve^8^ (Table 1).

**Table 1:** The severity of Parry–Romberg syndrome is divided into mild, moderate and severe.

Severity	Description
Mild	Skin and subcutaneous tissue atrophy limited to a single sensory branch of the trigeminal nerve
Moderate	Atrophy limited to 2 branches of the trigeminal nerve
Severe	Atrophy in a distribution involving all 3 branches of the trigeminal nerve or any bone involvement

The etiology and mechanism of the disease are poorly understood, and the treatments mainly focus on the reconstruction of the atrophic site^9^. Parry–Romberg syndrome is often considered to be an autoimmune condition with the same disease spectrum as local scleroderma en coup de sabre^10,11^. Some scholars believe that the causes of the disease include local facial trauma, sympathetic nervous system overactivity/hypofunction, trigeminal nerve abnormalities, collagen-related vasculitis vascular disease, and bacterial or viral infections including *Borrelia burgdorferi*^10,11^. Even less research has been done on the genetic aspects of Parry–Romberg syndrome. Jenny et al. conducted next-generation RNA sequencing in patients with Parry–Romberg syndrome and healthy individuals (as control)^12^. The results revealed that patients had a unique proinflammatory gene expression profile, including the upregulation of *GFCSF3, ADAMTS4* and *IL24.* These patients underwent surgical treatments, and their postoperative molecular signatures in skin became more similar to those of healthy individuals. To date, however, there are no studies on the genetic aspects of family genes associated with Parry–Romberg syndrome.

In this study, we performed whole-exome sequencing of patients with Parry–Romberg syndrome and their parents, from which we detected variant genes and associated variant loci. The variant genes and loci detected by whole-exome sequencing were further validated by one-generation sequencing on the patient's twin sister. Then we annotated the variant genes in associated available databases as well as functional analysis. We believe that the study on the genetic aspects of the family lineage can provide some new molecular information about the disease in order to facilitate our further understanding of the disease mechanism.

## Methods

### Participants

This study was conducted in accordance with the guidelines of the Declaration of Helsinki for Human Research and was approved by the Ethics Committee of Shanghai Ninth People’s Hospital Affiliated to Shanghai Jiaotong University School of Medicine. Informed consent was obtained from all participants, and their rights to privacy were preserved. A 25-year-old woman was diagnosed with Parry–Romberg syndrome according to her clinical manifestations, which presented with typical progressive unilateral facial soft tissue atrophy. Her parents and twin sister had normal craniomaxillofacial development. This patient, her parents and sister were enrolled in this study. We collected 2 ml of peripheral venous blood from these participants for later genetic testing analysis.

### Whole-exome sequencing

Whole-exome sequencing was performed by Shanghai We-Health Biomedical Technology Co., Ltd. Genomic DNA from peripheral venous blood samples of patient and the parents were extracted using a blood DNA extraction kit (DP348-03, Tiangen, Shanghai, China). The total amount and quality of DNA were checked by Onedrop OD1000 spectrophotometer and agarose gel electrophoresis. Exon capture experiments were performed using the Twist Comprehensive Exome Kit and sequenced on a HiSeq 4000 sequencer (Illumina, San Diego, CA), generating paired-end 150 bp reads at a target depth of 100 ×. Raw sequence alignments were performed using Burrows–Wheeler Aligner (BWA) and SAMtools software, Picard was used to deduplicate data, and Genome Analysis Toolkit (GATK v3.70) was used for variant calling.

### Sanger sequencing

Based on the results of whole-exome sequencing, we verified the genes in which the variants occurred by Sanger sequencing in the patient’s twin sister. The process of Sanger sequencing mainly includes PCR amplification, PCR purification, cycle sequencing, sequencing purification, capillary electrophoresis and data analysis. Sanger sequencing was also performed by Shanghai We-Health Biomedical Technology Co., Ltd.

### Functional analysis of mutant genes

We searched the GeneCards^®^: the Human Gene Database (https://www.genecards.org/) for variant genes, annotated them and analyzed their functions. The analysis of genes mainly includes genomic locations, molecular function, phenotypes, subcellular locations, super-pathway, interacting proteins and biological process.

### Ethical standards

All procedures followed were in accordance with the ethical standards of the responsible committee on human experimentation (institutional and national) and with the Helsinki Declaration of 1975, as revised in 2008 (5). Informed consent was obtained from all patients for being included in the study.

## Results

This 25-year-old woman diagnosed with Parry–Romberg syndrome firstly presented with facial skin atrophy on one side at the age of 11 years. The symptoms of facial atrophy gradually worsened, during which various immune-related indicators, such as antinuclear antibodies, were tested and the results were not abnormal. The patient's other systems, such as heart and kidney, showed no significant abnormalities in the associated tests. In later stages, the patient developed facial atrophy of subcutaneous tissue, fat and muscle. The patient's facial symptoms progressed for 8–10 years before no further changes were evident.

**Figure 1:**
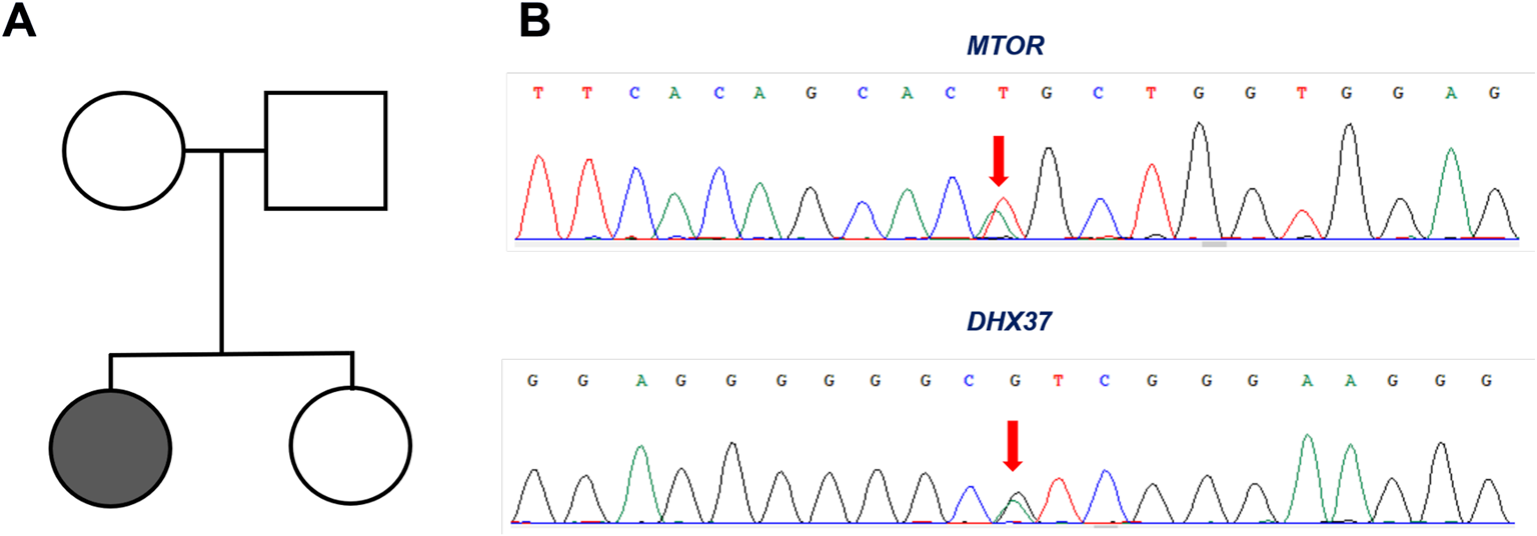
Variation characteristics of two gene loci. (**A**) The genetic map of the patient's family showed that there was only one case of Parry–Romberg syndrome in the patient's family. (**B**) The Sanger sequencing verification results of the patient's sister showed that the patient's sister also had the same genetic locus mutation.

**Figure 2:**
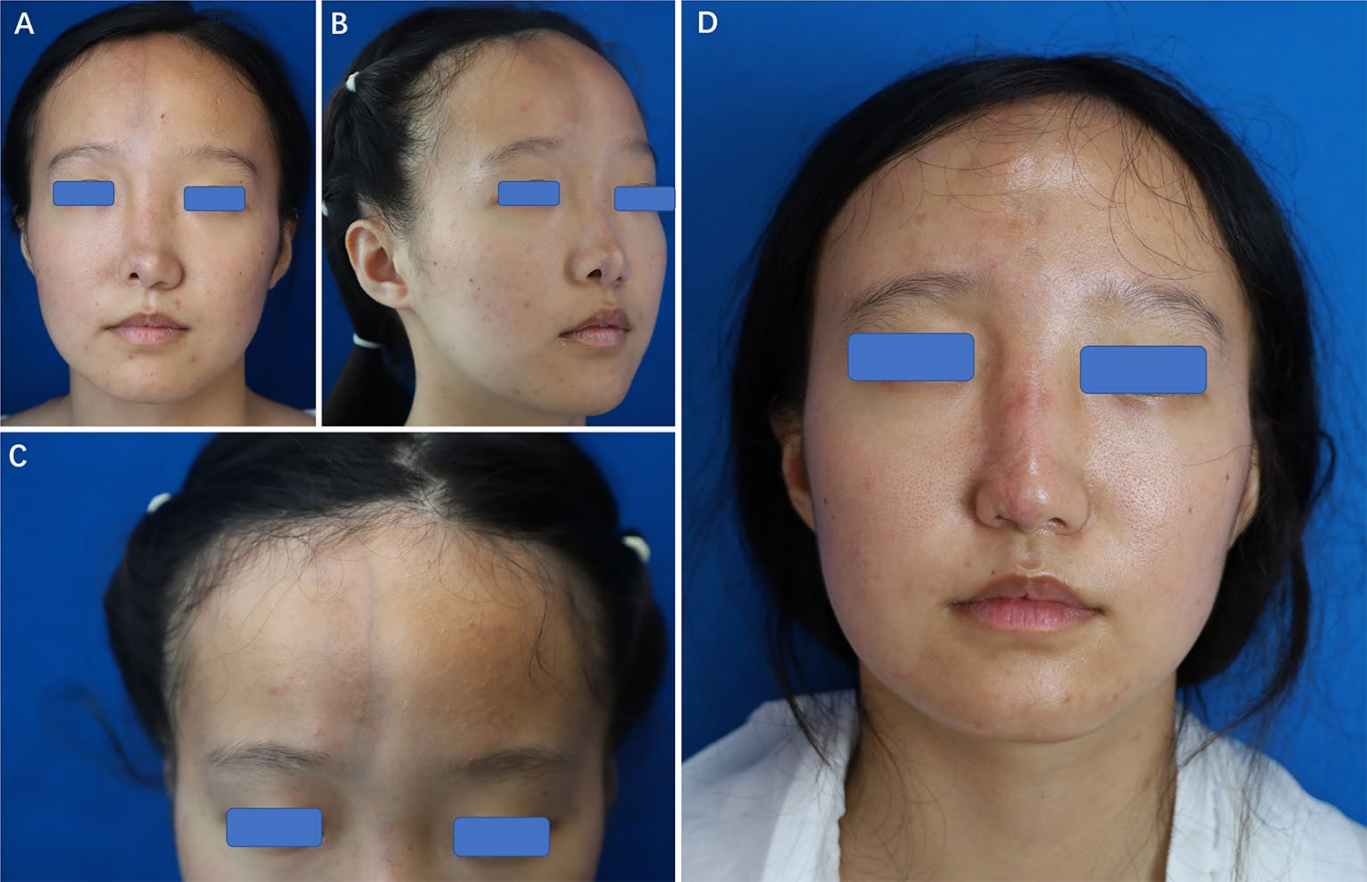
Photos of the patient's face before and after treatment. (**A**–**C**) Preoperative photos of patient. (**D**) Photo after two operations—autologous fat transplantation on the atrophic face and local flap transfer.

The genetic profile of the patient's family line is shown in Fig. 1A. In this family line, only the patient has the disease; the rest of the family, including the patient's twin sister, have a normal facial phenotype. The patient underwent two operations—autologous fat transplantation on the atrophic face, which resulted in some improvement in the atrophic face shape. Since the patient was concerned about the alar deformity, we performed local flap transfer to correct the alar deformity (Fig. 2).

The results of Whole-exome sequencing revealed that there were 2 variant loci in two genes, which were named *MTOR* and *DHX37*, respectively. The variant locus of *MTOR* was *MTOR*: NM_004958.4: exon31: c.4487A>T: p.Q1496L with dbSNP ID (rs1441021703). The variant locus of *DHX37* was *DHX37*: NM_032656.4: exon17: c.2180C>T: p.T727M with dbSNP ID (rs371186622). The variant frequencies of these two loci in the population were 0.000054 and 0.000367, respectively. Both variant genes were heterozygous in the patient. The variant *MTOR *was heterozygous in the patient’s father, while it was not detected in the patient’s mother. Similarly, the variant *DHX37 *was heterozygous in the patient’s mother, and it was not detected in her father.

As for the Sanger sequencing results, we validated the two variants in the patient's twin sister. The results of the test revealed that the patient's twin sister was heterozygous for both genetic variants of the locus (Fig. 1B). In other words, the patient's sister had the same two genetic variants as the patient. However, the facial clinical phenotype of the patient's sister was normal.

By searching the database, we annotated the variant genes as well as performed functional analysis. *MTOR* is short for mechanistic target of rapamycin kinase, and the protein encoded by this gene belongs to the phosphatidylinositol kinase-related kinase family. *DHX37* (DEAH-Box Helicase 37) encodes a DEAD box protein. DEAD box proteins are putative RNA helicases, which are characterized by the conserved motif Asp-Glu-Ala-Asp (DEAD). The genomic locations of *MTOR* and *DHX37 *are 1p36.22 and 12q24.31 (Supplementary Fig. 1). Phenotypes from GWAS catalog for *MTOR* and *DHX37* include 34 and 15 items, respectively. The associated phenotypes of these 2 genes are listed in Supplementary Table 1 and Supplementary Table 2. *MTOR* gene is widely expressed in various parts of the cell, and the top 3 subcellular locations for *MTOR* mainly include lysosome, cytosol and nucleus (Supplementary Fig. 2A). As to the subcellular locations for *DHX37* gene, they are focused on the nucleus (Supplementary Fig. 2B). There are 91 super-pathways for *MTOR* gene, of which the top 5 ones are RET signaling, Transcription Receptor-mediated HIF regulation, mTOR signalling, GAB1 signalosome and CD28 co-stimulation (Supplementary Table 3). For *DHX37* gene, there are only 2 associated super-pathways including rRNA processing in the nucleus and cytosol and Gene Expression (Supplementary Table 4).

**Figure 3:**
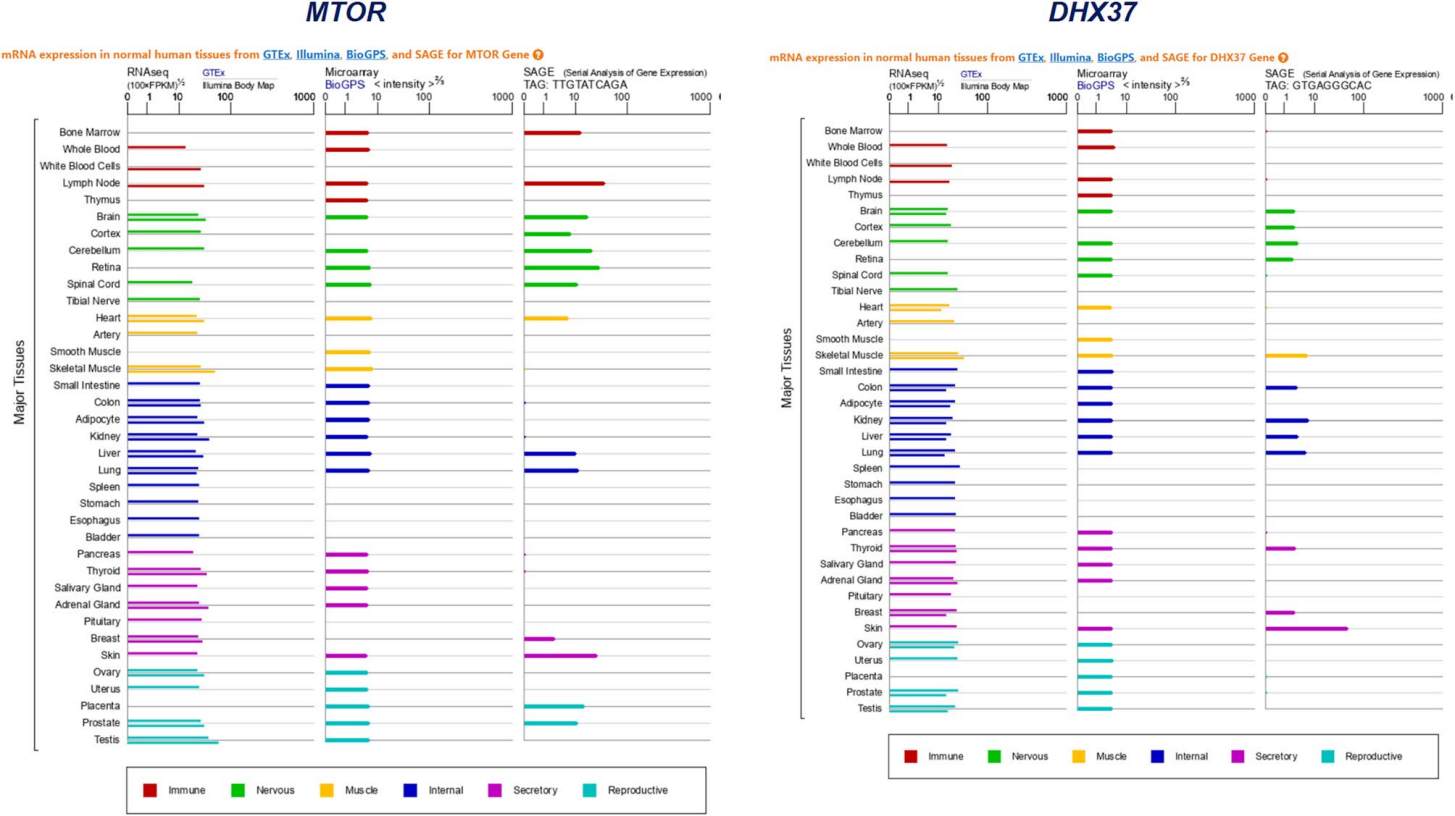
The mRNA expression in normal human tissues from GTEx, Illumina, BioGPS, and SAGE for *MTOR* and *DHX37* genes. The results showed high transcript levels of both genes in immune system, skin and skeletal muscle.

**Figure 4:**
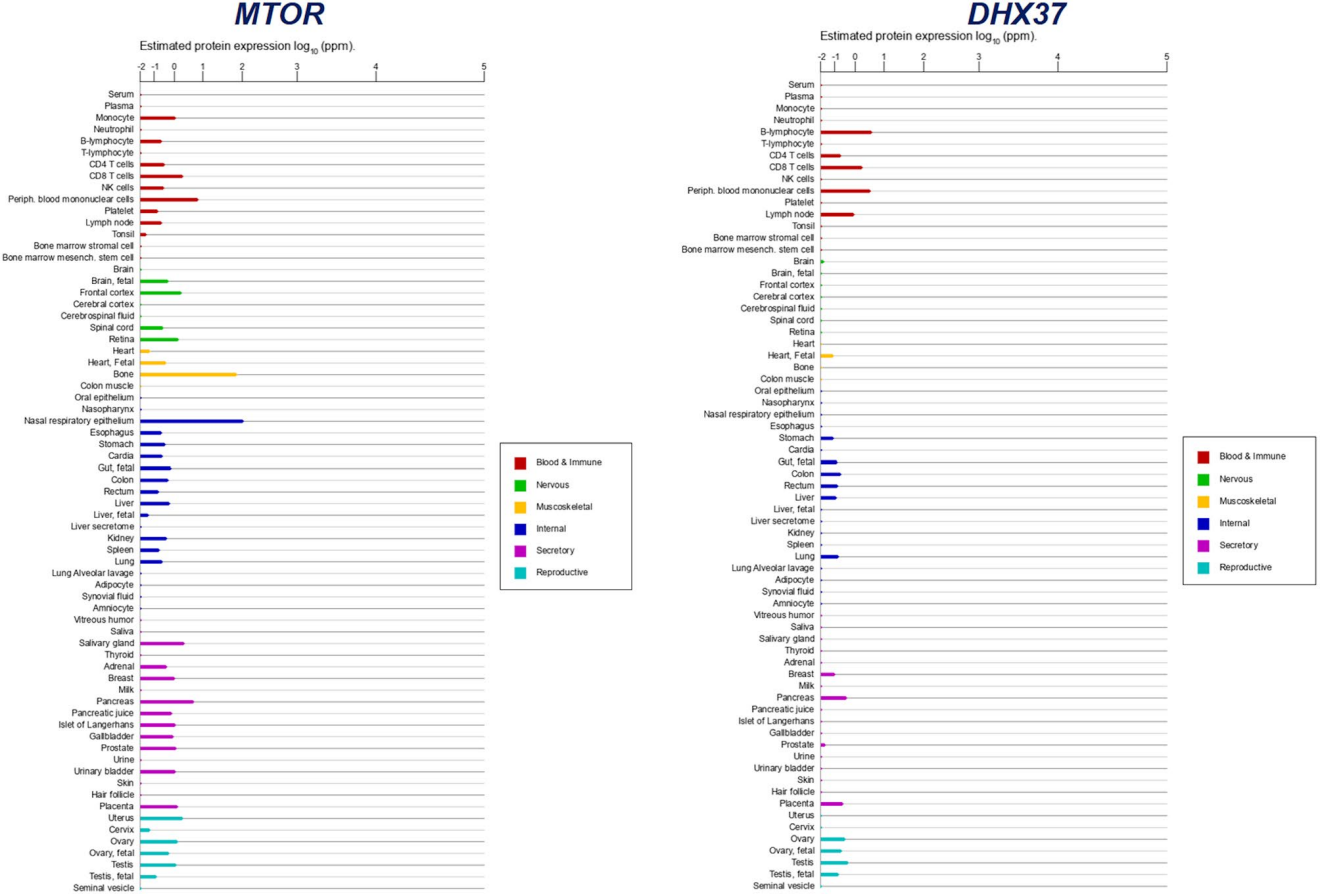
The protein expression levels in normal tissues and cell lines from ProteomicsDB, MaxQB, and MOPED for *MTOR* and *DHX37* genes. The results showed that the genes have a high level of protein expression in both blood and immune system.

**Figure 5:**
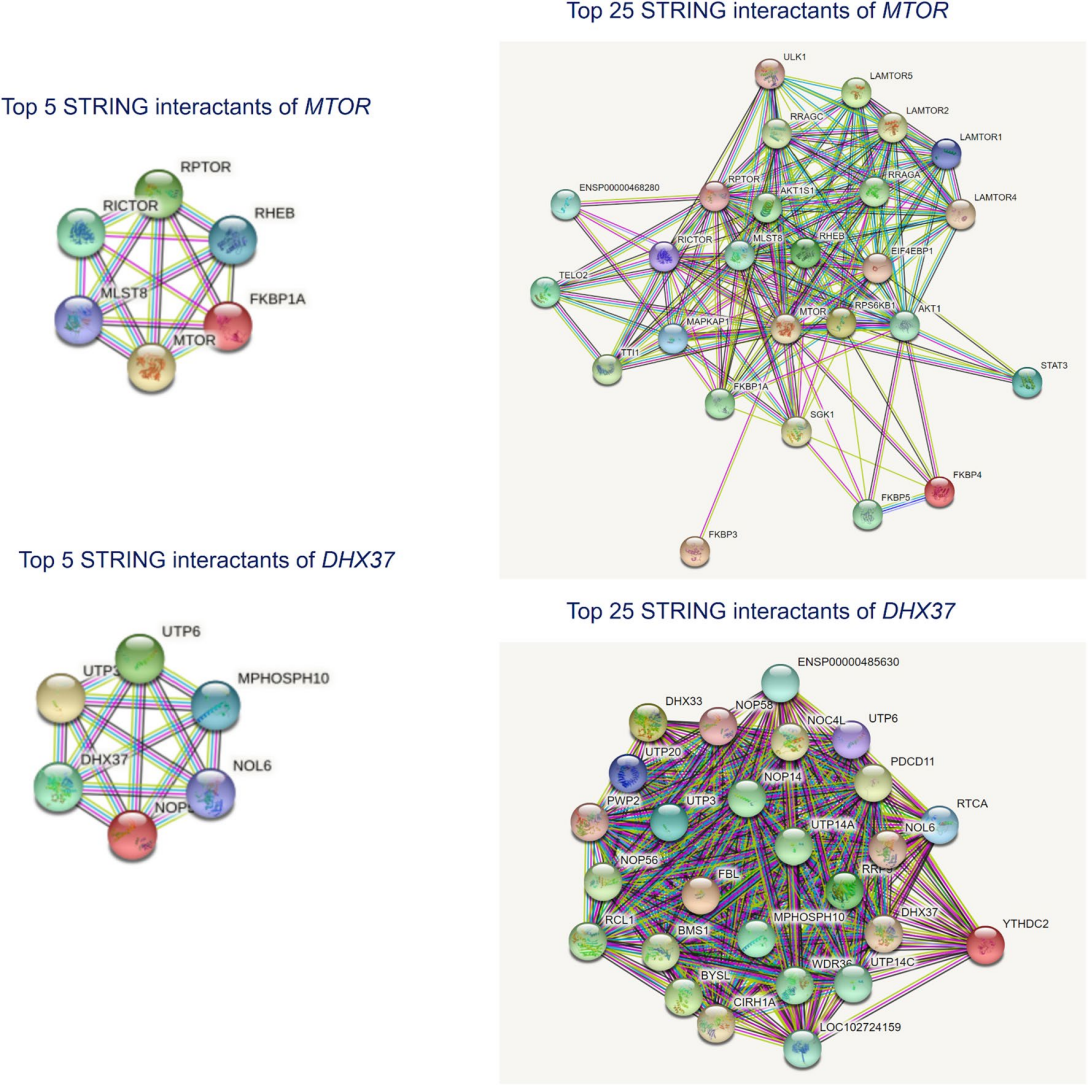
Interacting proteins analysis from STRING Interaction Network. The top 5 and 25 STRING interactants of both *MTOR* and *DHX37* genes are shown in this figure.

We also analyzed the expression of the 2 genes in normal human tissues. The mRNA expression in normal human tissues from GTEx, Illumina, BioGPS, and SAGE for *MTOR* and *DHX37* genes was shown in Fig. 3. The results showed high transcript levels of both genes in immune system, skin and skeletal muscle. As for protein expression levels, we searched related data in normal tissues and cell lines from ProteomicsDB, MaxQB, and MOPED for 2 genes. The results showed that the genes have a high level of protein expression in both blood and immune system (Fig. 4). Interacting proteins were analyzed from STRING Interaction Network. The top 5 STRING interactants of *MTOR* gene include* RPTOR, RHEB, RICTOR, MLST8 *and* FKBP1A. *The 5 STRING interactants of *DHX37* gene include *UTP6*, *UTP3*, *NOP56*, *NOL6* and *MPHOSPH10*. The top 25 STRING interactants of both genes were also concluded and the results of interacting proteins analysis are shown in Fig. 5.

## Discussion

Using Whole exome sequencing and Sanger sequencing, we examined genetic variants in patient with Parry–Romberg syndrome for the first time in a patient's family line. The detected variant loci were validated in the patient's twin sister. Our results suggest a new line of research for the correlation between genetic variants in Parry–Romberg syndrome and clinical phenotypes, that is, the detected loci variants may not lead directly to the clinical phenotype.

Parry–Romberg syndrome is a rare disease, the etiology and pathogenesis of which are not yet known^1,13^. There are few studies on genetic variants of the gene for Parry–Romberg syndrome^1,13^. Jenny et al. detected differentially expressed genes in patients' diseased tissues relative to normal subjects by second-generation sequencing of patient as well as normal human skin^12^. The sequencing results showed that there were 349 differentially up-regulated genes and 111 differentially down-regulated genes in the patient's lesioned tissue. Upregulated genes are enriched in various pathways including nuclear factor-κB in response to tumor necrosis factor, inflammation mediated by chemokines, cell proliferation and immune responses. The most highly up-regulated gene was *IL24*, which a member of the interleukin 10 family of cytokines. This gene is strongly associated with inflammatory and autoimmune diseases. Many down-regulated genes are evolutionarily conserved and implicated in orofacial development. The top 3 genes with the most significant down-regulation folds were *OPN1MW2*, *TRIM48*, and *C10orf96*, respectively. Jenny et al. also conducted an interesting study, in which they performed microvascular free tissue transfer to correct contour deformity in patient with Parry–Romberg syndrome. They found that gene expression in patients 6 months after surgery produced a large number of differential genes compared to pre-surgery, and that differential gene expression was significantly less in post-surgery patients compared to healthy controls than in pre-surgery. This result suggests that there is a tendency for patients' diseased tissues to shift toward normal tissues after surgery. Lyon et al. conducted whole exome sequencing of skin from the patients of Parry Romberg disease. Candidate genes were filtered using human phenotype ontology (HPO) terms specific to the Parry Romberg phenotype^14^. The results indicated that Candidate genes which may be implicated include *TCOF1*, *COL1A1* and *COL4A2*^14^. All of these studies provide valuable evidence for the development of genetic variation in hemifacial atrophy.

Most patients with hemifacial atrophy are sporadic, and few patients with the same disease in the same family have been reported. But Anderson reported two cases of hemifacial atrophy in the same family, one in a 9-year-old boy and the other in his cousin, a 14-year-old girl^15^. So it is not known whether hemifacial atrophy runs in families. The relationship between hemifacial atrophy and localized scleroderma is still controversial. There are common characteristics as well as differences between them. Lewkonia reported a case of hemifacial atrophy with localized scleroderma of the legs and trunk, and the serum in his system is positive for antinuclear antibodies^16^. These findings support the possibility that the disease is a variant of localized scleroderma rather than a developmental abnormality.

In this current surgery, two variant loci in two genes, which were named *MTOR* and *DHX37,* were detected and verified*.* Our conventional wisdom might suggest that the detected variant loci are usually directly responsible for the clinical phenotype of the patient. However, we performed Sanger validation on the patient's clinically phenotypically normal twin sister and found that the patient's sister also had consistent genetic variants. This suggests that the occurrence of the genetic variants may not necessarily lead to the development of an abnormal clinical phenotype in patients with Parry–Romberg syndrome. We searched the database for the mutated genes and analyzed their functions. We found high transcript levels of both genes in tissues such as skin and skeletal muscle, and the higher transcript levels in the immune system should also be of interest. At the protein translation level, we found that both 2 genes had high translation levels in the blood and immune system. These results can partially explain the atrophy of the patient's facial skin tissue, muscle tissue, etc. In addition, previous studies have suggested that the disease is an autoimmune disorder, while the results of the present study suggest a strong correlation between the mutated genes and the immune system, which is also consistent with previous studies.

The present study also has some limitations. First, this study included only one family member with hemifacial atrophy disease, which is a modest contribution to the study exploring the genetic type of the disease. The inclusion of more family lines could provide more basis for genetic type studies, and the inclusion of more family lines would allow more specimens to be collected for testing as a way to discover more and more precise information about genetic variants. However, because the disease is a rare disease, inclusion of more family lines does present a greater difficulty. In addition, we did not validate the patient's parents in the Sanger sequencing validation, which may reduce the confidence of the variant gene detection. However, the Sanger validation of the patient's sister could partially compensate for this aspect. Finally, we were not able to collect lesioned tissue specimens from patients, and further testing of lesioned tissue specimens for variant gene expression proteins could also increase the precision of this study.

## Conclusions

In the present study, two novel loci of *MTOR* and *DHX37 *genes were detected in a family of patient with Parry–Romberg syndrome, which were not reported in previous studies. These two genes have a strong correlation with the immune system, which is consistent with previous reports that the disease is an autoimmune disorder. The patient's twin sister had the same genetic locus variants but did not exhibit the abnormal clinical trait of hemifacial atrophy, which suggests these variants may not directly determine the clinical phenotype. When studying clinical etiology, other factors, such as the environment, should also be taken into account.

## Supplementary Information

Supplementary Figure 1.

Supplementary Figure 2.

Supplementary Tables.

Supplementary Legends.

## Data Availability

The datasets used and/or analyzed during the current study are available from the corresponding author on reasonable request.
