# Supplementary figures and images for: Genetic variations in patient with Parry–Romberg syndrome
Source: Sci Rep. 2023 Jan 9;13:400. doi: 10.1038/s41598-023-27597-1 (PMC9829853; doi:10.1038/s41598-023-27597-1)

## *MTOR*

Chr 1

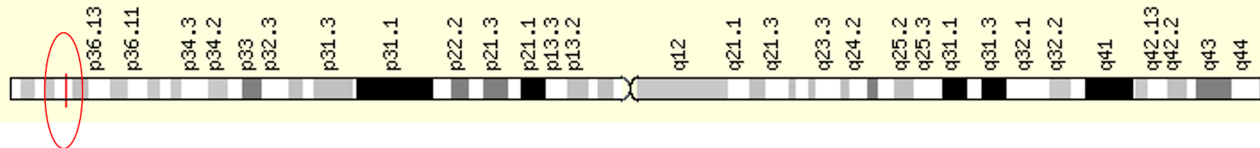

## *DHX37*

Chr 12

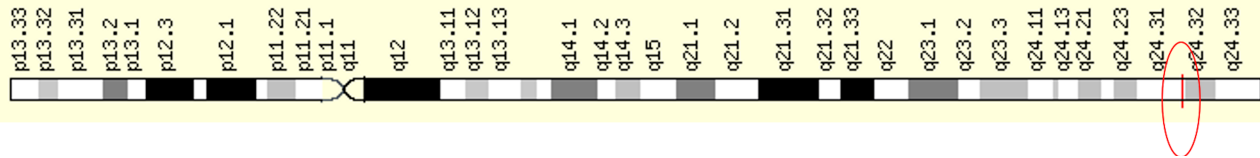

Supplement: Supplementary file 1 — Supplementary Figure 1. [file 41598_2023_27597_MOESM1_ESM.pdf]
