# Supplementary material for: Genetic variations in patient with Parry–Romberg syndrome
Source: Sci Rep. 2023 Jan 9;13:400. doi: 10.1038/s41598-023-27597-1 (PMC9829853; doi:10.1038/s41598-023-27597-1)
Supplement: Supplementary file 2 — Supplementary Figure 2. [file 41598_2023_27597_MOESM2_ESM.pdf]

MTOR

A

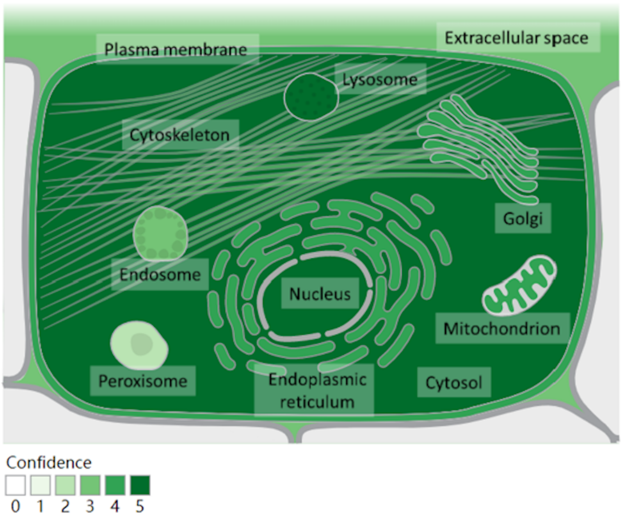

Compartment	Confidence
lysosome	5
cytosol	5
nucleus	5
golgi apparatus	4
endoplasmic reticulum	4
mitochondrion	4
plasma membrane	4
endosome	3
cytoskeleton	3
extracellular	3
peroxisome	2

DHX37

B

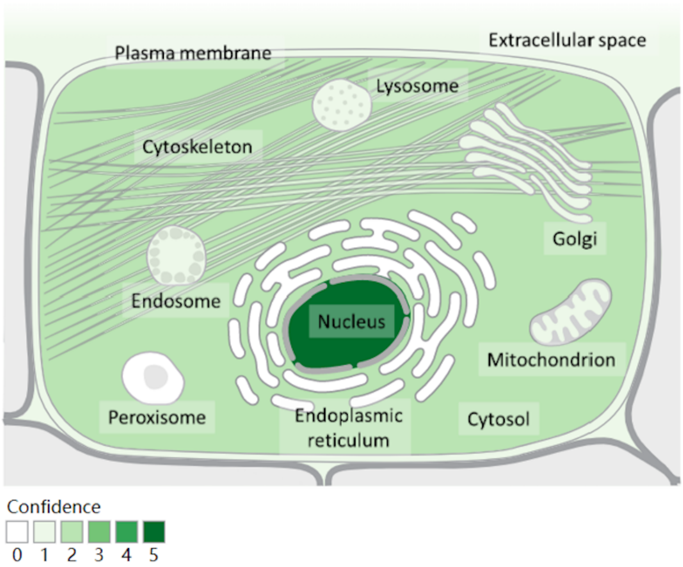

Compartment	Confidence
nucleus	5
cytosol	2
golgi apparatus	1
lysosome	1
endosome	1
mitochondrion	1
cytoskeleton	1
extracellular	1
plasma membrane	1
