# Supplementary material for: Genetic variations in patient with Parry–Romberg syndrome
Source: Sci Rep. 2023 Jan 9;13:400. doi: 10.1038/s41598-023-27597-1 (PMC9829853; doi:10.1038/s41598-023-27597-1)
Supplement: Supplementary file 3 — Supplementary Tables. [file 41598_2023_27597_MOESM3_ESM.docx]

**Supplementary Table 1.** Phenotypes from GWAS catalog for *MTOR.*

| Number | Phenotype | Gene Relation | Best Score | Mean Score | # of Snps | # of Studies | SNP IDs |
| --- | --- | --- | --- | --- | --- | --- | --- |
| 1 | Varicose veins | GeneHancer | 103.0 | 81.0 | 1 | 3 | rs11121615 |
| 2 | corneal topography | GWAS | 46.0 | 23.5 | 3 | 3 | rs17036350  rs3737611  rs74225573 |
| 3 | balding measurement | GWAS,  GeneHancer | 41.7 | 24.5 | 2 | 2 | rs143353512  rs184689679 |
| 4 | eosinophil count | GeneHancer | 32.0 | 24.9 | 1 | 3 | rs35249183 |
| 5 | body height | GWAS | 26.1 | 26.1 | 1 | 1 | rs2300092 |
| 6 | sex hormone-binding globulin measurement | GWAS,  GeneHancer | 21.7 | 13.3 | 2 | 2 | rs1884429  rs75077113 |
| 7 | mean corpuscular volume | GWAS | 21.0 | 13.0 | 2 | 2 | rs112659347  rs75077113 |
| 8 | heel bone mineral density | GWAS | 20.7 | 14.0 | 2 | 3 | rs2261434  rs75077113 |
| 9 | intraocular pressure measurement | GWAS | 20.5 | 20.5 | 1 | 1 | rs28991009 |
| 10 | resting heart rate, chronic obstructive pulmonary disease | GeneHancer | 17.4 | 17.4 | 1 | 1 | rs59985166 |
| 11 | eosinophil percentage of leukocytes | GeneHancer | 16.7 | 14.5 | 1 | 2 | rs35249183 |
| 12 | monocyte count | GeneHancer | 15.7 | 15.7 | 1 | 1 | rs284324 |
| 13 | body weight | GWAS,  GeneHancer | 15.5 | 10.5 | 2 | 2 | rs2788543  rs77270747 |
| 14 | mean reticulocyte volume | GWAS | 15.3 | 15.3 | 1 | 1 | rs112659347 |
| 15 | leukocyte count | GeneHancer | 15.0 | 11.5 | 2 | 2 | rs284316  rs284317 |
| 16 | red blood cell density measurement | GeneHancer | 14.4 | 13.0 | 2 | 1 | rs284312  rs6540943 |
| 17 | appendicular lean mass | GWAS,  GeneHancer | 12.7 | 11.2 | 2 | 1 | rs1034528  rs11121615 |
| 18 | serum alanine aminotransferase measurement | GeneHancer | 12.5 | 12.5 | 1 | 1 | rs743577 |
| 19 | basophil count, eosinophil count | GeneHancer | 12.0 | 12.0 | 1 | 1 | rs35249183 |
| 20 | body mass index | GWAS,  GeneHancer | 11.7 | 9.1 | 8 | 5 | rs1010447  rs10779751  rs11581010  rs1205593  rs1884429  rs2791643  rs284262  rs544722 |
| 21 | lean body mass | GeneHancer | 11.7 | 9.6 | 1 | 1 | rs1884429 |
| 22 | blood urea nitrogen measurement | GeneHancer | 10.7 | 8.1 | 2 | 2 | rs4845945  rs74748843 |
| 23 | erythrocyte count | GeneHancer | 10.4 | 10.4 | 1 | 1 | rs7523471 |
| 24 | eosinophil percentage of granulocytes | GeneHancer | 10.0 | 10.0 | 1 | 1 | rs35249183 |
| 25 | aspartate aminotransferase measurement | GWAS,  GeneHancer | 10.0 | 9.3 | 2 | 2 | rs4845860  rs743577 |
| 26 | glomerular filtration rate | GeneHancer | 9.5 | 9.1 | 2 | 2 | rs284316  rs74748843 |
| 27 | cystatin C measurement | GeneHancer | 9.3 | 9.3 | 1 | 1 | rs1884429 |
| 28 | neutrophil count | GeneHancer | 8.7 | 8.7 | 1 | 1 | rs578347 |
| 29 | electrocardiography | GeneHancer | 8.5 | 8.1 | 1 | 1 | rs578347 |
| 30 | BMI-adjusted waist circumference | GWAS | 8.3 | 8.3 | 1 | 1 | rs7553827 |
| 31 | facial morphology measurement | GeneHancer | 8.2 | 8.2 | 1 | 1 | rs143353512 |
| 32 | facial hair thickness measurement | GWAS | 6.4 | 6.4 | 1 | 1 | rs11121667 |
| 33 | gut microbiome measurement | GeneHancer | 5.7 | 5.7 | 1 | 1 | rs146609044 |
| 34 | acute myeloid leukemia | GeneHancer | 5.1 | 5.1 | 5 | 1 | rs11121613  rs17035680  rs17035686  rs205488  rs6540948 |

**Supplementary Table 2.** Phenotypes from GWAS catalog for *DHX37.*

| Number | Phenotype | Gene Relation | Best Score | Mean Score | # of Snps | # of Studies | SNP IDs |
| --- | --- | --- | --- | --- | --- | --- | --- |
| 1 | BMI-adjusted waist-hip ratio | GeneHancer | 70.4 | 41.6 | 4 | 2 | rs10773051  rs10773052  rs11057418  rs7296124 |
| 2 | BMI-adjusted waist circumference | GeneHancer | 50.0 | 38.8 | 2 | 2 | rs10773051  rs11057418 |
| 3 | high density lipoprotein cholesterol measurement | GeneExon,  GWAS,  GeneHancer | 39.4 | 25.4 | 2 | 4 | rs4078216  rs67053123 |
| 4 | BMI-adjusted hip circumference | GeneHancer | 20.5 | 13.8 | 3 | 1 | rs10773051  rs11057418  rs825452 |
| 5 | heel bone mineral density | GeneHancer | 15.0 | 15.0 | 1 | 1 | rs825453 |
| 6 | mean corpuscular hemoglobin concentration | GeneHancer | 12.0 | 10.8 | 1 | 1 | rs825453 |
| 7 | lipoprotein measurement, blood metabolite measurement | GeneHancer | 12.0 | 12.0 | 1 | 1 | rs67053123 |
| 8 | total cholesterol measurement | GeneHancer | 11.7 | 10.0 | 1 | 2 | rs67053123 |
| 9 | body height | GeneHancer | 11.4 | 9.3 | 2 | 2 | rs13624  rs143270813 |
| 10 | hair colour measurement | GeneHancer | 7.7 | 7.7 | 1 | 1 | rs77717551 |
| 11 | body mass index | GeneHancer | 7.3 | 7.3 | 1 | 1 | rs825452 |
| 12 | cocaine dependence | GeneHancer | 6.3 | 6.3 | 1 | 1 | rs150954431 |
| 13 | insulin measurement, glucose tolerance test | GeneExon,  GWAS | 5.5 | 5.5 | 1 | 1 | rs150152903 |
| 14 | hip circumference | GeneExon,  GWAS | 5.4 | 5.4 | 1 | 1 | rs150152903 |
| 15 | COVID-19 | GeneHancer | 5.4 | 5.4 | 1 | 1 | rs150954431 |

**Supplementary Table 3.** Top 5 super-pathways for *MTOR* gene.

| Number | SuperPathway | Contained pathways | |
| --- | --- | --- | --- |
| 1 | RET signaling | Downstream signal transduction  0.94  Signaling by PDGF  0.94  DAP12 signaling  0.92  Insulin receptor signalling cascade  0.92  Signaling by Insulin receptor  0.92  IRS-mediated signalling  0.91  Signaling by EGFR  0.90  IGF1R signaling cascade  0.90  IRS-related events triggered by IGF1R  0.90 | Signaling by Type 1 Insulin-like Growth Factor 1 Receptor (IGF1R)  0.90  DAP12 interactions  0.89  NGF signalling via TRKA from the plasma membrane  0.87  Signaling by SCF-KIT  0.85  Signalling by NGF  0.81  Fc epsilon receptor (FCERI) signaling  0.79  VEGFA-VEGFR2 Pathway  0.78  Signaling by VEGF  0.76 |
| 2 | Transcription Receptor-mediated HIF regulation | Regulation of lipid metabolism Insulin signaling-generic cascades  0.59  Translation Insulin regulation of translation  0.59  Transcription Receptor-mediated HIF regulation  0.51  Translation Regulation of EIF4F activity  0.44 | Development CNTF receptor signaling  0.43  Signal transduction PTEN pathway  0.39  Development Growth hormone signaling via PI3K/AKT and MAPK cascades  0.34  ErbB2/ErbB3 signaling events  0.31 |
| 3 | mTOR signalling | Energy dependent regulation of mTOR by LKB1-AMPK  0.72  mTOR signalling  0.72  PKB-mediated events  0.70  mTORC1-mediated signalling  0.56 | PI3K Cascade  0.51  Target Of Rapamycin (TOR) Signaling  0.38  Macroautophagy  0.30 |
| 4 | GAB1 signalosome | PIP3 activates AKT signaling  0.91  Role of LAT2/NTAL/LAB on calcium mobilization  0.91  GAB1 signalosome  0.89 | PI3K/AKT activation  0.89  PI3K/AKT Signaling in Cancer  0.72 |
| 5 | CD28 co-stimulation | CD28 co-stimulation  0.67  CD28 dependent PI3K/Akt signaling  0.67 | Costimulation by the CD28 family  0.45  VEGFR2 mediated vascular permeability |

**Supplementary Table 4.** Super-pathways for *DHX37* gene.

| Number | SuperPathway | Contained pathways | |
| --- | --- | --- | --- |
| 1 | rRNA processing in the nucleus and cytosol | Major pathway of rRNA processing in the nucleolus and cytosol  0.94  rRNA processing in the nucleus and cytosol  0.94 | rRNA processing  0.89  rRNA modification in the nucleus and cytosol  0.33 |
| 2 | Gene Expression | Gene Expression  0.48 |  |
