# Supplementary material for: Genetic variations in patient with Parry–Romberg syndrome
Source: Sci Rep. 2023 Jan 9;13:400. doi: 10.1038/s41598-023-27597-1 (PMC9829853; doi:10.1038/s41598-023-27597-1)
Supplement: Supplementary file 4 — Supplementary Legends. [file 41598_2023_27597_MOESM4_ESM.docx]

**Supplementary Figure 1.** The genomic locations of *MTOR* and *DHX37* (1p36.22 and 12q24.31, respectively)*.*

**Supplementary Figure 2.** The subcellular locations for *MTOR* and *DHX37* genes. **A**, *MTOR* gene is widely expressed in various parts of the cell, and the top 3 subcellular locations for *MTOR* mainly include lysosome, cytosol and nucleus. **B**, as to the subcellular locations for *DHX37* gene, they are focused on the nucleus.
